# Seasonal patterns in stable isotope and fatty acid profiles of southern stingrays (*Hypanus americana*) at Stingray City Sandbar, Grand Cayman

**DOI:** 10.1038/s41598-020-76858-w

**Published:** 2020-11-12

**Authors:** Lisa A. Hoopes, Tonya M. Clauss, Nicole E. Browning, Alexa J. Delaune, Bradley M. Wetherbee, Mahmood Shivji, Jessica C. Harvey, Guy C. M. Harvey

**Affiliations:** 1 Department of Research and Conservation, Georgia Aquarium, Atlanta, GA 30313 USA; 2 Department of Animal Health, Georgia Aquarium, Atlanta, GA 30313 USA; 3 Department of Mathematics and Science, Brenau University, Gainesville, GA 30501 USA; 4 Department of Biological Sciences, University of Rhode Island, Kingston, Rhode Island 02881 USA; 5 The Guy Harvey Research Institute, Halmos College of Natural Sciences and Oceanography, Nova Southeastern University, Dania Beach, Florida USA; 6 Guy Harvey Ocean Foundation, Grand Cayman, KY1-1005 Cayman Islands

**Keywords:** Ecology, Zoology

## Abstract

Ecotourism opportunities in the marine environment often rely heavily on provisioning to ensure the viewing of cryptic species by the public. However, intentional feeding of wildlife can impact numerous aspects of an animals’ behavior and ecology. Southern stingrays (*Hypanus americana*) provisioned at Stingray City Sandbar (SCS) in Grand Cayman have altered diel activity patterns and decreased measures of health. This study looked at seasonal changes in stable isotope (SI) and fatty acid (FA) profiles of provisioned stingrays at SCS. Plasma δ^15^N was higher in male stingrays (11.86 ± 1.71‰) compared to females (10.70 ± 1.71‰). Lower values for δ^15^N in males and females were measured in October during low tourist season, suggesting stingrays may be forced to rely on native prey items to supplement the decreased amount of provisioned squid available during this time. Plasma FA profiles were significantly different between sexes and across sampling time points, with FAs 22:6n3, 16:0, 20:5n3, 18:1n3C, 18:0 and 18:1n9T contributing to dissimilarity scores between groups. Dietary FAs primarily contributed to differences between males and females lending further evidence to differences in foraging patterns at SCS, likely due to intraspecific competition. Further, canonical analysis of principal coordinates (CAP) analysis of FA profiles suggest similar diets during peak tourist season and differences in diet between males and females during the low season. This study demonstrates alterations in feeding ecology in stingrays at SCS which is of critical importance for effective management of the SCS aggregation.

## Introduction

Ecotourism that consists of observing wildlife is a thriving business that generates billions of dollars to local economies^1–3^. Non-consumptive wildlife based tourism has been well established in terrestrial settings, where opportunistic viewing of species is likely^4^, however, marine ecotourism is continually gaining popularity^1^. The marine environment presents challenges to wildlife viewing as species spend most of their time below the surface, out of sight of hopeful tourists. In order to maximize the chances of encounters with marine species, tour operators often rely more heavily on provisioning^5^. Although intentional feeding of wildlife can impact movement patterns, population size, reproduction, behavior, and health of these animals, many wildlife interaction operations continue the practice, often with unmeasured consequences^6^. The distribution of food resources is one of the most important factors shaping behaviors of wild animals^7^, and not surprisingly, the provisioning of marine species greatly influences the behavior of marine wildlife^6,8^. Provisioning of marine wildlife at tourist interaction sites often involves providing non-native prey items in unnatural quantities that affects the condition of fed animals^6,9^. Despite the large-scale changes to diet, rate of consumption, and overall feeding ecology of marine wildlife that results from provisioning at tourist interaction sites, few of these features have been investigated^4,10,11^.

Feeding ecology of marine species is difficult to ascertain due to foraging primarily occurring out of sight, and indirect measures of diet and foraging are more commonly utilized^12^. Historically, methodologies such as direct observations, stomach contents and fecal analyses have been utilized, often in combination^13^. While these approaches are useful, each have limitations. For example, limited visibility will bias observational data. Similarly, stomach contents of predators will be over-represented by prey that contain hard and indigestible parts that are retained in the stomach and under-represented by easily digested prey that remain in the stomach for shorter periods of time^14,15^. These techniques only provide a snapshot of feeding and do not provide long term information on feeding ecology. Two indirect methods that are commonly used to gain insight into the long-term feeding ecology of animals are stable isotope (SI) analysis and fatty acid (FA) profile analyses^16–18^, both of which have been used extensively in feeding ecology studies of marine species (e.g.,^19–21^). When used together, SI and FA profile analyses can be powerful tools in identifying differences/similarities in feeding ecology among and between species, between sexes, and across spatial and temporal scales (e.g.,^22–24^).

Stable isotope analysis is based on measuring naturally occurring isotopes (e.g., C, N, S, O) that are passed from prey to predator and are reflected in the tissues of the consumer. Lighter isotopes are typically preferentially metabolized, leaving the heavier isotope to increase in concentration, resulting in a predictable, step-wise enrichment called fractionation (also known as diet-tissue discrimination factors^25,26^). Nitrogen and carbon are commonly used in feeding ecology studies where δ^15^N indicates trophic level position of the consumer, and δ^13^C, which reflects the source of primary producers in food webs, provides information about habitat use of the consumer^16,17,25, 27,28^.

Fatty acid profile analysis is based on a similar “you are what you eat” premise^29^, but this technique has the potential to evaluate feeding on a finer scale compared to SI analysis. Fatty acids are components of lipids and consumers can synthesize some FAs de novo, while others can only be obtained from the diet. These essential FAs derived from the diet can be used as dietary biomarkers^30^. In marine food webs, long chain FAs pass from prey to carnivore consumers relatively unchanged, allowing for the reflection of a prey’s FA profile to be represented in the tissues of the consumer^31^. This is particularly true of fish who lack the ability to biosynthesize long-chain polyunsaturated FAs (PUFA), and thus, must rely on their naturally PUFA-rich diet to obtain these essential FAs^32,33^.

Southern stingrays inhabit tropical and subtropical waters of the Western Atlantic Ocean from New Jersey to southern Brazil^34,35^. They are generalist and opportunistic nocturnal foragers, feeding on a variety of benthic decapod crustaceans and teleosts, and to a lesser extent, on molluscs and annelids^36–38^. Historical studies of diet for this species have primarily been limited to the examination of stomach contents. Southern stingrays are popular animals for tourist-wildlife interactions, usually involving provisioning. One such locale is Stingray City Sandbar (SCS), Cayman Islands, where southern stingrays are found year-round in dense aggregations consisting of both sexes at a naturally occurring sandbar in the North Sound lagoon of Grand Cayman^39^. This aggregation of stingrays is a long-standing ecotourism attraction where supplemental feeding of these animals has been ongoing since 1986^40,41^. Although both sexes occur at SCS, the aggregation of stingrays is comprised of approximately 80% female, most of which are mature^39^. Previous dietary studies at SCS have shown that stingrays are fed squid (*Illex* and *Loligo* sp.), a non-native diet item sourced from the North Atlantic and North Pacific, by tourists nearly every day from early morning to mid-afternoon as tour boats deliver tourists to the SCS site^6,42,43^. This heavy reliance on provisioning by stingrays at SCS has resulted in altered blood FA profiles compared to non-tourist fed stingrays sampled in Grand Cayman^42^.

Acoustic tracking of provisioned stingrays at SCS revealed that they were continuously active throughout the day moving among tourists offering food, but moved little at night in contrast to increased nocturnal movement and daytime resting exhibited by wild stingrays^6^. However, differences in activity budgets between provisioned males and females were noted, with tagged males ranging further along the fringing reef at night compared with females who remained stationary at the feeding site. Corcoran et al.^6^ suggested that males may forage at night to supplement an inadequate amount of food received during the daytime at SCS as they are outcompeted for food at SCS by females, which tend to be larger and receive more food from tourists^39^. Provisioning of stingrays with a non-native diet is central to maintaining the presence of animals at SCS and the economic value of SCS to the local community is estimated at $50 million USD, annually (Cayman Islands Department of Environment, personal communication). This, in combination with recent fluctuations in stingray aggregation levels at SCS, highlights the need to understand the feeding ecology of these stingrays. The goal of this study was to assess fine scale temporal and sex differences in the feeding ecology of stingrays at the SCS using stable isotopes and fatty acids as non-invasive biomarkers of diet.

## Results

The female stingrays randomly selected for the study averaged 107.2 cm disc width (DW), while the males were considerably smaller, as expected with this sexually dimorphic species (53.8 cm DW). All animals had a PIT tag at the initial capture in January (10 males, 10 females) and once it was discovered that re-capture rate in April was poor for males (20% re-capture rate of males [2 individuals], 18 females), animals were sampled opportunistically in the subsequent months of July (7 males, 18 females), and October (7 males, 17 females).

### Stable isotopes

**Figure 1:**
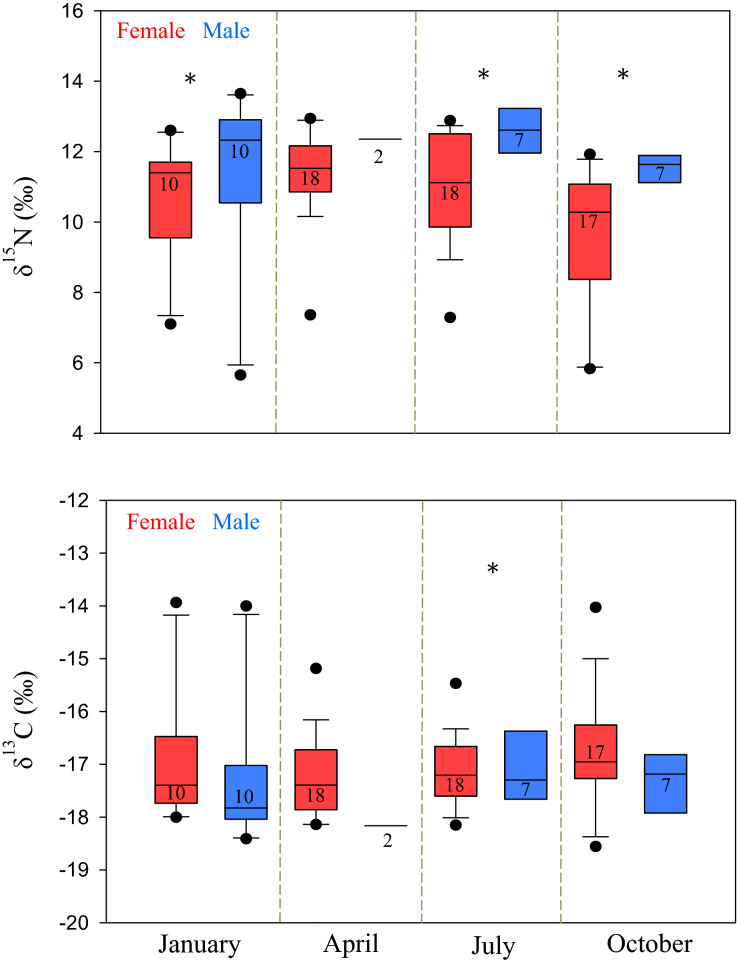
Box plots of δ^15^N and δ^13^C plasma isotopes for female and male stingrays sampled at SCS in January, April, July, and October. Significant differences between sexes within a sampling month are denoted by an asterisk (*) and numbers represent sample size. This figure was created with SigmaPlot 12.5 (Systat Software, San Jose, CA).

**Figure 2:**
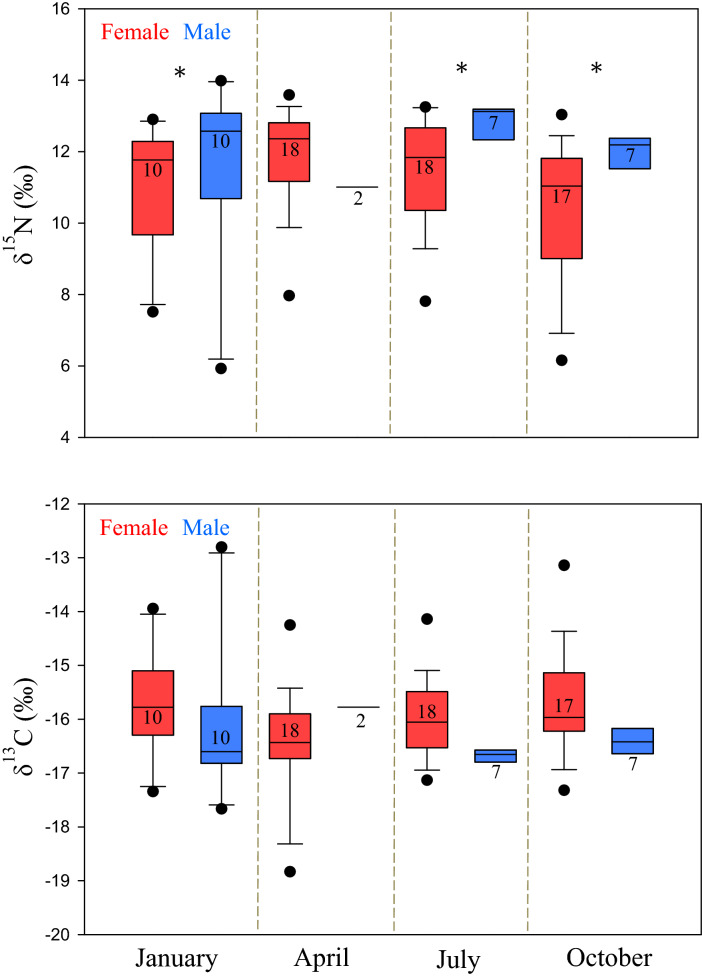
Box plots of δ^15^N and δ^13^C whole blood isotopes for female and male stingrays sampled at SCS in January, April, July, and October. Significant differences between sexes within a sampling month are denoted by an asterisk (*) and numbers represent sample size. This figure was created with SigmaPlot 12.5 (Systat Software, San Jose, CA).

Differences between sexes and among the sampling time points were observed for δ^15^N and δ^13^C values in both plasma (Table 1, Fig. 1) and red blood cells (Table 1, Fig. 2). Model coefficients for the plasma samples indicated significant differences in δ^15^N between sexes (χ^2^_1,89_ = 4.05, *p* = 0.044, n = 89) and across sampling points (χ^2^_3,89_ = 65.6, *p* < 0.001, n = 89). δ^15^N averaged 11.9 ± 1.7‰ for males and 10.7 ± 1.7‰ for females. Pairwise comparisons of the population by month show that the lowest values of δ^15^N in plasma were from October (10.2 ± 1.9‰) which was significantly lower than values from January (11.0 ± 2.0‰, *p* = 0.002, n = 44), April (11.5 ± 1.2‰, *p* < 0.001, n = 44), and July (11.4 ± 1.5‰, *p* < 0.001, n = 49). The interaction between sex and sampling point was also significant (χ^2^_3,89_ = 26.8, *p* < 0.001, n = 89), with males in January (*p* = 0.001, n = 89), July (*p* < 0.001, n = 89), and October (*p* < 0.001, n = 89) displaying greater plasma δ^15^N compared to females (Fig. 1).

**Table 1:** Mean (± SD) δ^15^N and δ^13^C (‰) measured in the plasma and red blood cells of male and female southern stingrays sampled in January, April, July, and October at Stingray City Sandbar. Sample sizes in parentheses.

	January		April		July		October	
**Plasma**								
δ^15^N Male	11.4 ± 2.5	(10)	12.4 ± 0.1	(2)	12.6 ± 0.9	(7)	11.6 ± 1.1	(7)
δ^15^N Female	10.6 ± 1.7	(10)	11.4 ± 1.3	(18)	11.0 ± 1.5	(18)	9.7 ± 2.0	(17)
δ^13^C Male	−17.3 ± 1.4	(10)	−18.2 ± 0.1	(2)	−17.0 ± 0.8	(7)	−17.4 ± 1.0	(7)
δ^13^C Female	−17.0 ± 1.2	(10)	−17.2 ± 0.8	(18)	−17.1 ± 0.7	(18)	−16.7 ± 1.1	(17)
**Red blood cells**								
δ^15^N Male	11.7 ± 2.5	(10)	11.0 ± 0.3	(2)	12.9 ± 0.8	(7)	12.0 ± 0.9	(7)
δ^15^N Female	11.0 ± 1.7	(10)	11.9 ± 1.4	(18)	11.4 ± 1.5	(18)	10.3 ± 1.9	(17)
δ^13^C Male	−16.1 ± 1.5	(10)	−15.8 ± 0.2	(2)	−16.7 ± 0.2	(7)	−16.3 ± 0.4	(7)
δ^13^C Female	−15.7 ± 0.9	(10)	−16.5 ± 1.0	(18)	−16.0 ± 0.7	(18)	−16.3 ± 0.4	(17)

No significant differences were detected in plasma δ^13^C between males and females (χ^2^_1,89_ = 0.30, *p* = 0.585, n = 89), however, values differed significantly among sampling time points (χ^2^_3,89_ = 16.4, *p* < 0.001, n = 89). Pairwise comparisons indicated that δ^13^C was higher in October (-16.9 ± 1.1‰) compared with July (−17.1 ± 0.7‰, *p* = 0.066, n = 49, this is considered significant given the conservative nature of the Šidák correction for multiple comparisons^44^). Differences between males and females across sampling points were significant (χ^2^_3,89_ = 13.8, *p* = 0.003, n = 89) for the month of July (*p* = 0.001) when the plasma of males was higher in δ^13^C compared to females (Fig. 1).

Model coefficients for red blood cells showed δ^15^N values did not differ significantly between sexes (χ^2^_1,89_ = 1.42, *p* = 0.233, n = 89), but did differ significantly among sampling time points (χ^2^_3,89_ = 13.5, *p* = 0.004, n = 89). Pairwise comparisons indicated higher δ^15^N in red blood cells in July (11.9 ± 1.5‰) compared with values from October (10.8 ± 1.9‰, *p* = 0.010, n = 49). The interaction between sex and sampling point was significant (χ^2^_3,89_ = 8.71, *p* = 0.033, n = 89). Significant differences in δ^15^N in red blood cells were observed between males and females in January (*p* = 0.019, n = 89), July (*p* = 0.004, n = 89), and October (*p* = 0.008, n = 89) (Fig. 2). Differences in δ^13^C were not significant between sexes (χ^2^_1,89_ = 0.025, *p* = 0.614, n = 89) or sampling time points (χ^2^_3,89_ = 1.04, *p* = 0.791, n = 89), and the interaction between the two was also not significant (χ^2^_3,89_ = 0.029, *p* = 0.961, n = 89).

### Fatty acids

A total of 52 FAs were identified in plasma samples, with 37 FAs identified at levels greater than 0.1% (Table S1). Stingray plasma was proportionally dominated by polyunsaturated FAs (36.6%), and saturated FAs (33.5%), with lower levels of monounsaturated FAs (11.5%). Branched chain FAs were not measured. Dominant fatty acids (> 5%) in decreasing order of relative importance included: 16:0, 22:6n3 (docosahexaenoic acid, DHA), 20:5n3 (eiscosapentaenoic acid, EPA), and 18:0.

There were significant differences in FA profiles of stingrays among sampling points (pseduo-F = 6.90, *p*(perm) < 0.001) and between sexes (pseudo-F = 2.90, *p*(perm) = 0.049). Differences in FA profiles between sexes (17.7% dissimilarity, SIMPER) were primarily in FAs 22:6n3, 16:0, 20:5n3, 18:1n3C, 18:0, and 18:1n9T. The average dissimilarity (SIMPER) between any two combinations of samples (either between sexes or among sampling time points) ranged from 13.5–22.8%, with FAs 22:6n3, 16:0, 20:5n3, 18:1n9C and 18:0 accounting for the majority of differences among groups. FAs that contributed to at least 50% of the cumulative total difference (dissimilarities 9.1–16.8%) between males and females within months were 22:6n3, 16:0, 20:5n3, 18:0, 18:1n9C, 18:2n6, 18:3n3, 18:1n7, 24:0, 18:1, 20:1n11, 14:0 (SIMPER), with contributions of individual FAs differing based on the various combinations of sex and month. These FAs were included in further multivariate analyses using CAP. Despite the variation in contribution of the individual FAs to the overall dissimilarity distance between sexes at each sampling point, average abundance scores for dietary FAs 22:6n3 and 20:5n3 consistently contributed (7–12% and 6–8%, respectively) to these differences.

**Figure 3:**
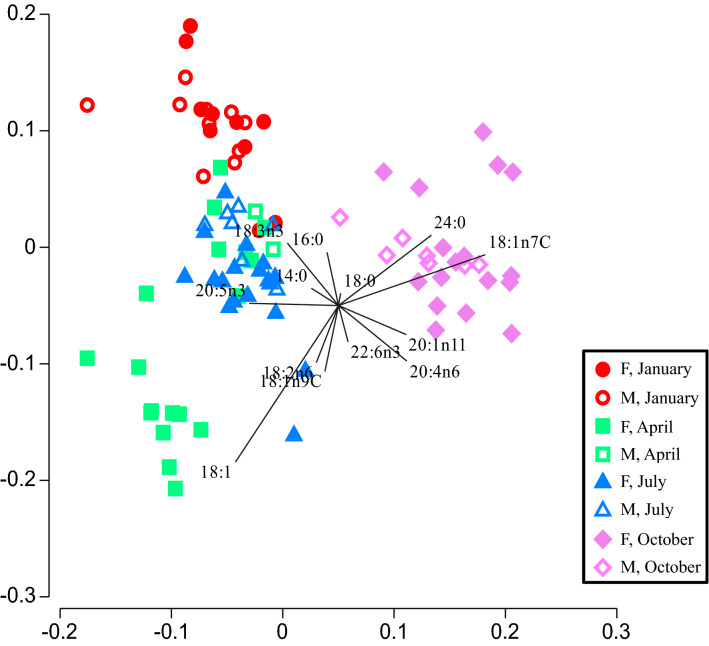
Canonical analysis of principle coordinates (CAP) of male (M) and female (F) stingrays within each sampling time point (January, April, July, October) with overlaid vectors of individual fatty acids identified by SIMPER routines. This figure was created with PRIMER/PERMANOVA + version 7 (Primer-e Ltd, Aukland, New Zealand).

Males and females within sampling month were distinguished by FA profiles (CAP *p* = 0.001). Fatty acids identified by SIMPER routines were overlaid as vectors on the CAP ordination plot (Fig. 3).

## Discussion

Few studies have explored stable isotopes in species belonging to the Family *Dasyatidae.* Nitrogen isotope compositions in the muscle of brown stingrays *Dasyatis lata* in Kane’ohe Bay, Hawai’i (range δ^15^N 10.4 to 11.9‰, δ^13^C −13.5 to −11.9‰^45^) were similar to those found in southern stingrays in our study (range δ^15^N 9.7 to 12.6‰, δ^13^C -18.2 to -16.7‰ in plasma). However, muscle sampled from southern stingrays in Belize had lower δ^15^N values (mean δ^15^N 7.4‰ and δ^13^C -8.5‰^46^), which the authors suggested may have resulted from a diet of soft-bodied invertebrates and bivalves rather than crabs and teleost fish typically consumed by southern stingrays in other locations^37^. δ^13^C values for southern stingrays at SCS were lower compared to values reported for brown stingrays in Hawaii and southern stingrays in Belize and differences likely reflect different habitats and sources of carbon at the base of each food web^16^.

**Figure 4:**
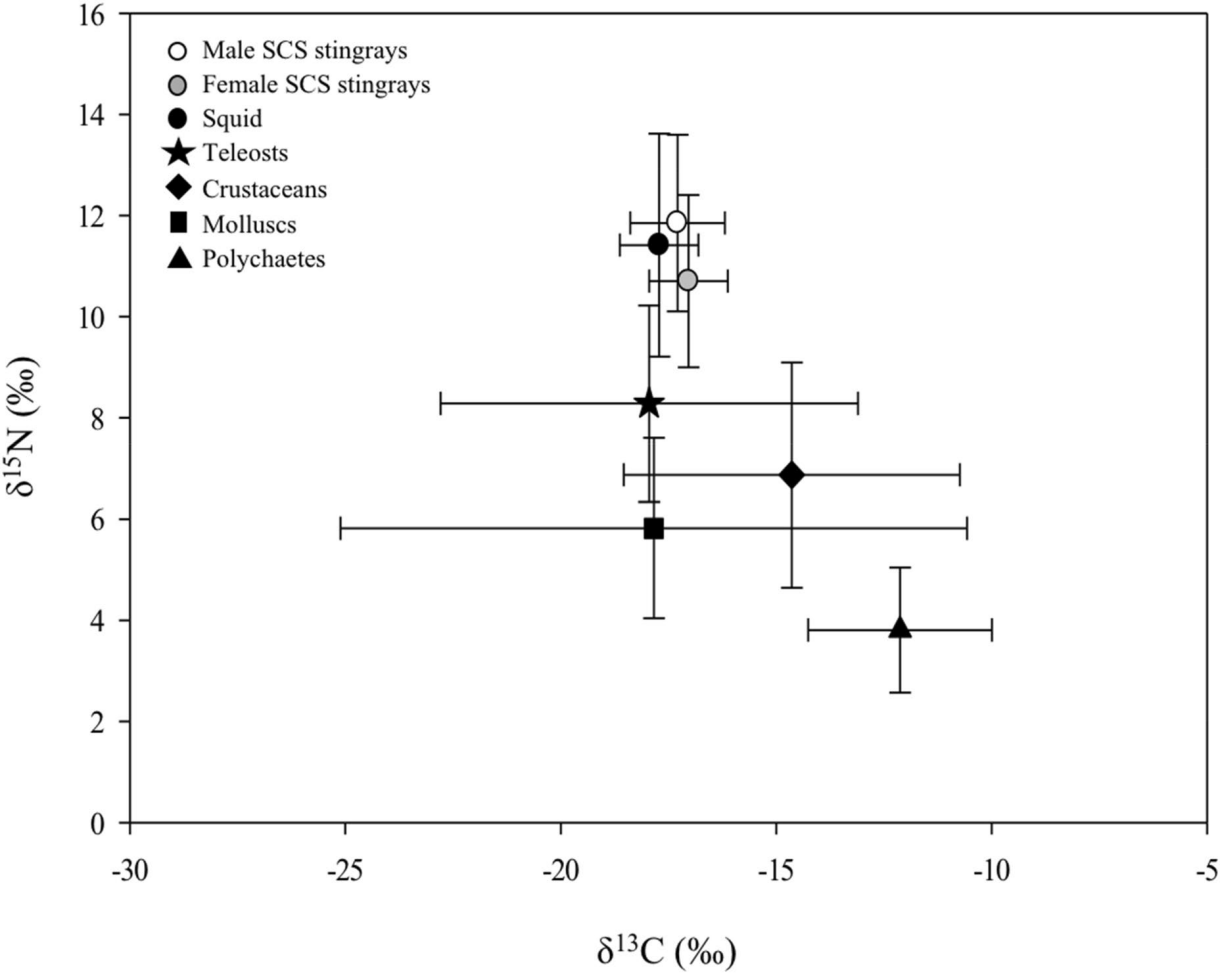
Mean (±SD) plasma δ^13^C and δ^15^N from male (white circle) and female (grey circle) southern stingrays at Stingray City Sandbar and potential primary prey groups (black symbols) as derived from the literature. This figure was created with SigmaPlot 12.5 (Systat Software, San Jose, CA).

The heavy nitrogen isotope (^15^N) increases in concentration in a stepwise fashion with increases in trophic level. Conversely, δ^13^C is conserved through trophic systems, and δ^13^C values are predominantly used to determine the source of carbon from primary producers^16,17,47^. Fractionation values differ for different species and tissues sampled and few studies have measured fractionation values in elasmobranchs. Only a single study has examined fractionation values in batoid rays. Controlled feeding trials conducted on smallnose fanskates (*Sympterygia bonapartii*) resulted in fractionation values of 1.7 ± 0.1‰ for δ^13^C and 2.5 ± 0.2‰ for δ^15^N in whole blood ^48^. Plasma of male stingrays at SCS was found to be higher in δ^15^N compared to females (11.9 ± 1.7‰ vs. 10.7 ± 1.7‰) in our study and males had significantly higher δ^15^N than females year-round, except in April. Lack of a difference between the sexes in April likely reflects the small number of males that were captured (n = 2). Similar trends were observed for δ^15^N values in red blood cells, where males had higher δ^15^N compared to females in all months with the exception of April. Higher δ^15^N in males may be indicative of males feeding at a higher trophic level (although fractionation values do not indicate a full trophic level increase) than females, nutritional stress in males, differences in growth rates between males and females and/or differences in diet quality, both of which can influence δ^15^N discrimination values^49^. Squid offered at the SCS was not sourced locally and therefore may have δ^15^N values that do not reflect the regional food web. Based on stomach content analyses^36–38,50,51^, stable isotope values of potential prey of southern stingrays from the US, Bahamas, Belize, and Cuba, were extracted from the literature (Table S2) and plotted with squid values (*Illex* and *Loligo* sp.) from the North Pacific and North Atlantic (Fig. 4) to understand what may be driving these differences in nitrogen signatures between males and females at SCS.

Higher δ^15^N in the plasma and red cells of males in this study suggests males may be receiving more provisioned squid in their diet than females. This is unexpected given the differences in movement patterns of male and female stingrays at the SCS^6^ and despite a clear preference for the provisioning of larger females by tour operators (L. Hoopes, personal observation). Corcoran et al.^6^ noted that males moved actively along reef edges at night while females remained inactive in close proximity to SCS. They suggest that larger females, who were targeted by tour operators, were receiving greater quantities of squid and out-competing males for access to provisioned squid, thus requiring them to engage in nocturnal foraging^6^. Similar competition between sexes for tourist-derived food was observed in Australia where large female *Dasyatis* stingrays behaved aggressively towards smaller males^52^. If males were foraging nocturnally to supplement their diet with natural prey items, their diet would likely be comprised of crustaceans (portunid crabs, penaeid shrimp) and teleost fishes (Families Labridae, Gobiidae, Scaridae) which accounted for 87% of stomach contents of southern stingrays in the Bahamas^37^. However, δ^15^N values of native prey from the literature range broadly from 4 to 8‰, at least one trophic level below the values sampled in males in this study (Fig. 4). Potential native prey was not sampled during this study and SI values for invertebrates and teleosts within the Cayman Islands could not be found in the literature. While prey values from other locations may not accurately reflect baseline δ^15^N within SCS and surrounding reef systems, male stingrays would need to be foraging on a native diet at an unrealistically high trophic level to reflect the blood nitrogen isotope composition measured in this study. Non-provisioned stingrays were not able to be sampled as controls in this study and we cannot eliminate the possibility that differences in δ^15^N between males and females were an artifact of variables not related to provisioned feeding.

Elevated δ^15^N signatures can be indicative of nutritional stress in the form of caloric restriction, fasting, or starvation^53–56^. During periods of nutritional stress, animals may catabolize their own ^15^N-rich tissues (essentially feeding at a higher trophic level) causing an increased excretion of ^15^N-depleted nitrogeneous wastes, resulting in tissues elevated in ^15^N^17^, including short-term increases in blood δ^15^N^53,57,58^. Studies attempting to measure higher δ^15^N during nutritional stress have been equivocal (e.g.,^55,59^). Logan and Lutcavage^60^ measured temporal increases in blood δ^15^N in skates (*Leucoraja* sp.) who lost weight due to poor eating in the control group of a 20-d experimental turnover trial. Conversely, Wyatt et al.^61^ noted a reduction in δ^15^N in the plasma of whale sharks (*Rhincodon typus*) fasting 151 days and suggested that urea may have an influence on tissue isotopes in calorically limited animals, although this individual later died and may have had other complicating health factors. Visual assessment of body condition of male stingrays during handling did not indicate starvation (L. Hoopes, personal observation) and higher δ^15^N in males due to severe caloric restriction or starvation seems unlikely.

Differences in growth rates can affect stable isotope turnover and tissue discrimination and higher growth rates reduce nitrogen discrimination^62^. While male southern stingrays do exhibit faster growth rates than females in this sexually dimorphic species^39^, animals sampled in this study were within reported ranges for maximum disc width for adult males and females. This suggests that these animals had reached maturity were not likely actively growing and δ^15^N values would not be greatly affected by this variable.

Variation in δ^15^N may reflect differences in diet quality. In this context, diet quality is defined as the amino acid composition of a food source relative to the needs of a consumer^63^, but should also consider proximate composition (protein, fat, and carbohydrate balance) and the vitamins and trace minerals that are available across a range of prey items. Generally, carnivores feeding across a diversity of prey items would be expected to have high quality diets since they tend to consume prey with similar protein and amino acid compositions to their own tissues^61^. Higher quality diets result in dietary amino acids being routed directly to tissues with little processing and thus low isotopic discrimination. Conversely, lower quality diets (low protein with highly imbalanced amino acid compositions compared with consumer requirements) can inflate fractionation values of ^15^N compared to high quality diets since consumers would need to synthesize scarce amino acids de novo from surplus amino acids^49,63^. As secondary consumers, squid (*Ilex* and *Loligo* sp.) have crude protein levels within the range of other teleosts (L. Hoopes, unpublished data), however, amino acid compositions are variable with notable differences in the proportion of glycine, proline, and arginine in squid compared to other marine prey^64^. It is unclear if the amino acid composition of a predominately squid diet meets stingray dietary requirements compared to a more varied diet including potential native prey at lower trophic positions, but it is unlikely that isotopic differences between males and females reflect greater de novo synthesis of certain amino acids. Regardless, the reduction in isotopic discrimination in higher order carnivores remains and area where further study is warranted^65^ and future studies using compound specific amino acid stable isotope analysis may be useful in understanding whether diet quality differences exist between males and females feeding at SCS.

To evaluate the temporal scale in which isotopic values reflect diet, the turnover rates of those tissues must be known^61^. Isotopic turnover rate is the amount of time it takes for a given consumer tissue to incorporate the isotopic composition of the diet. Turnover rates vary depending on species, type of tissue being sampled, metabolic rate and animal size, and environmental variables like water temperature^61,66–70^. Assessing turnover time requires controlled feeding studies which are challenging to conduct, as they require long study periods and isotopically distinct diets^71^. Results of the few studies on turnover rates in elasmobranch species indicate that for plasma, the estimated days to 50% incorporation (half-life) of the new isotopic signature is approximately 35–60 days, although, whale sharks appear to have longer blood turnover rates (90–200 d)^48,60,61,72,73^.

δ^15^N was significantly lower in October compared to other months in both red blood cells and plasma, indicating a probable shift in the diet of both males and females. October coincides with low numbers of tourists visiting the SCS (Port Authority of the Cayman Islands, www.caymanport.com), presumably leading SCS stingrays to supplement their diets during this time feeding on a greater proportion of wild prey. Diet studies are lacking for southern stingrays from Grand Cayman, therefore using stable isotope values to infer consumption of specific prey must be interpreted cautiously. Lower δ^15^N signatures in invertebrate and teleost prey from the literature, compared to δ^15^N values in squid (Fig. 4) could suggest a greater reliance on native prey items during the low tourist season as SCS.

There were no differences in δ^13^C between males and females across tissues for any sampling month, with the exception of the month of July when plasma in males was slightly higher in δ^13^C (mean 0.12‰) than females. Carbon isotope compositions can become higher as a predator moves from feeding in an offshore food web to an inshore source^74^, however, these animals have shown high levels of site fidelity around the SCS site^6^ and these detected differences are probably not biologically significant.

Fatty acid profiles for stingray plasma were significantly different across the year and between sexes and SIMPER results consistently identified dietary FAs (22:6n3, 18:1n9C, 20:5n3) contributing to these patterns. Fatty acid profiles have previously been examined in this population of stingrays^43^, and direct comparisons are not possible because of inconsistencies in the units reported. However, percent 20:5n3 (EPA) and 22:6n3 (DHA) reported by Semeniuk et al. (^42^, 10.1% and 25.5% respectively) were similar to values obtained in our study (6.9% EPA and 22.6% DHA, respectively), suggesting a continuing pattern of squid provisioning at this SCS site almost a decade later. Fatty acid profiles of southern stingrays feeding in the south sound of Grand Cayman on a native diet had lower levels of both 20:5n3 and 22:6n3 (4.7% and 12.7% respectively^42^). The authors attributed the higher 20:5n3 and 22:6n3 values in SCS stingrays (along with lower 20:4n6 values) to the provisioned squid diet when compared to similar levels in conspecifics feeding on a native diet^42^. Low levels of dietary FAs 20:5n3 and 22:6n3 were also measured in southern stingrays collected in the Gulf of Mexico^75^.

Fatty acid values in closely related potential prey species have been measured and show lower levels of 22:6n3 compared to those in squid, further supporting the inference that changes in FA values reflect a shift in diet during parts of the year. For example, portunid crab species have 22:6n3 levels between 8.5 and 17.1% compared to 34 to 43% in squid^76–80^. Levels of 22:6n3 in parrotfish (livers) are reported as 3.2% ^81^. Lower levels of 22:6n3 are also reported in mantis shrimp (10.4 to 15.1%^82,83^), in various species of marine annelids (2.6 to 5.2%^84^) and in conch species (2 to 5%, L. Hoopes unpublished data,^84^).

While higher δ^15^N in males suggested greater consumption of provisioned squid compared to females, finer scale dietary changes were detected in FA profiles suggesting some scope for dietary differences (11–24%, SIMPER) between males and females depending on season. Fatty acid profiles for males and females in October were different from stingrays sampled at other times of the year (Fig. 4), with FAs 18:1n7C, 24:0, 20:1n11 and 20:4n6 being the primary drivers of these differences. FAs 18:1n7 and 20:1n11 have been shown to be indicators of copepod-consuming species like crustaceans, bathypelagic squid, and fish in the diets of deepwater elasmobranch species^85,86^. While 18:1n7 can be metabolized from 16:1n7, Beckmann et al.^87,88^ found this FA to be a reliable marker during controlled feeding trials with Port Jackson sharks (*Heterodontus portusjacksoni*). Monounsaturated FA 20:1n11 is typically found in high levels in cold water fish species^18,31^, and in lower levels in tropical and subtropical fish^89^, however, median levels of this FA were not significantly different across sampling time points. High levels of FA 20:4n6 (arachidonic acid, 13%) have been associated with Port Jackson sharks feeding on macro-algae consuming invertebrates (sea urchins and sea snails), while piked dogfish (*Squalus megalops*) known to feed exclusively on cephalopods had lower (4.6%) tissue levels of 20:4n6^90^. Conversely, Semeniuk et al.^42^ noted higher levels of 20:4n6 in the plasma of native feeding southern stingrays (11.5%) compared to provisioned squid eating animals at SCS (5.2%). The incorporation of FAs from prey into the tissues of the consumers can be a complex process to understand in the elasmobranchs, however, the individual FAs driving these differences may suggest more reliance on native prey items in October compared to other months. October is considered the low season for tourists at SCS (Port Authority of the Cayman Islands, www.caymnaport.com) and if tourist-provided squid are not as plentiful, foraging for wild prey may be increased.

Conversely, FAs 20:5n3 and 16:0 were drivers of FA profiles in stingrays sampled in January and July (Fig. 3). Fatty acid 20:5n3 has been previously used as a biomarker of diatom-based food webs^33^ and controlled feeding trials in Port Jackson sharks show it to be a more accurate indicator of diet in blood, compared to dietary FA 22:6n3^88^. Semeniuk et al.^42^ noted high proportions of 20:5n3 in cold water elasmobranchs and stingrays at SCS fed a non-native diet of squid (25.5%). Fatty acids 20:5n3, 22:6n3, 18:1n9 and 18:2n6 have previously been identified as useful indicators of diet in the liver and muscle of deepwater chrondrichthyans^85^ and in the liver, muscle, and blood of Greenland sharks (*Somniosus microcephalus*^33^). January is considered the height of tourist season at SCS and visitor numbers to the sandbar reach yearly highs (Port Authority of the Cayman Islands, www.caymanport.com). This may represent a time in which provisioning is at its peak and ostensibly, squid are plentiful for both male and female stingrays. Curiously, during the month of January, male and female FA profiles differed by 24% (SIMPER) suggesting there may be some finer scale seasonal dynamics at play within this population.

Although FA assimilation in sharks and rays appears to be similar to other taxa^87,88^, elasmobranchs rely on ketone bodies instead of lipids for energy metabolism and rely on lipid-rich livers for long-term energy storage^91^. The influence of caloric restriction and/or fasting on plasma fatty acids signatures in sharks is unknown. Wood et al.^92^ noted decreases in non-esterified FAs in the plasma of dogfish sharks (*Squalus acanthias*) after feeding compared to fasted control animals, but they did not examine individual FAs and further research is warranted. The functions and fates of individual fatty acids during elasmobranch feeding and fasting may be taxa-specific and controlled feeding studies would further enhance the quantitative capacity of FA profiling in sharks and rays^93^.

Stable isotope data suggests a consistent, long-term pattern of provisioning was occurring at the SCS tourist site, however, FA profiles of SCS stingrays suggests small dietary differences between males and females during certain parts of the year, although natural temporal or spatial variations cannot be ruled out. Precise driving factors of these dietary differences remain unclear, but appear to be related seasonal differences in the quantity of squid available at the SCS and the nature of feeding on natural prey away from the SCS. Similar FA profiles between sexes in January suggest that both males and females are relying on provisioned squid during the height of tourist season, but when the number of tourists visiting SCS declines (October), both sexes may consume a larger proportion of natural prey items. Larger sample sizes for males as well as a SI and FA database of potential wild prey items would provide additional information for explaining feeding history and for predator–prey comparisons of profiles using Bayesian mixing models and quantitative fatty acid signature analysis^18^. Decades of provisioning have altered the feeding ecology of stingrays at SCS and considering the great economic value of the aggregation of stingrays, understanding the long-term effects of provisioning on the biology and overall health of the stingrays is of critical importance for well-informed and effective management of the SCS aggregation to ensure continued success of interaction activities.

## Methods

This study was conducted with approval from the Cayman Islands Government Department of Environment. Animal handling and sampling was approved through the Research Committee at Georgia Aquarium and all handling procedures were conducted using guidelines established by the American Fisheries Society and American Society of Ichthyology and Herpetology.

### Study site

The SCS is a small, shallow sandbar (7800 m^2^) with minimum depth of 0.5 m and sandy bottom located in the North Sound of Grand Cayman (19°22′34″N, 81°18′18″W) (Fig. S1). North of the sandbar is an adjacent patchwork of deeper reefs and habitat to the south is primarily seagrass beds. There are over one million visitors to the SCS site annually, where they interact with approximately 100 stingrays, 80% of which are large mature females (personal communication Cayman Islands Department of Environment,^39^). Stingrays at SCS show very high fidelity to the feeding site, with females resident for an average of 10 years, and males resident for an average of 3 years^39^, demonstrating chronic provisioning of the animals.

### Sampling and handling

Ten female and ten male stingrays ranging in disc width from 55 to 121 cm were chosen at random in January 2014 for this study. All animals had an existing passive integrated transponder (PIT) tag suggesting they were a known resident of the SCS population. Subsequent 4-day trips in April, July, and October of 2014 attempted to locate and re-sample these same 20 individuals, with mixed success. Between January and April, re-capture success of the original 10 females was 80%, while only 2 of the original 10 males were able to be located and re-sampled. Based on this low re-capture rate of males (20% re-capture in April), we opportunistically sampled males, and additional females, the remainder of the study period.

Stingrays were captured with hand-held dip nets and transferred into a seawater-filled pool inside a boat for examination and sample collection as part of a larger study. Once transferred onboard, handling time averaged 11 min and consisted of scanning for the presence of PIT tags, standard morphometrics, blood sampling, ultrasound assessment, and PIT tagging of any new individuals. Upon completion, stingrays were placed back in the dip net and returned to the water. Visual observation of released stingrays indicated that they typically resumed feeding within several minutes of being returned to the water from nearby tourist feeding interactions.

Blood was collected either ventrally from the caudal tail vein (21 g × 1.5 in. needles) or dorsally from a pectoral fin vessel (21 g or 23 g × 1 in. butterfly needle) and then transferred into lithium heparin vacutainers. No difference in blood isotope or fatty acid profiles were expected between venipuncture sites based on similarities in blood chemistry values from paired samples (L. Hoopes, unpublished data). Blood volume ranged from 3–12 ml depending on the size of the animal. Samples were kept chilled until they were able to be centrifuged at 3300 rpm for 10 min, 1–3 h post-collection. Separated plasma and spun red cells were aliquoted to cryovials and stored at −80 °C in a charged, dry nitrogen shipper (MVE Series DOBLE-20, New Prague, MN) until analyzed. Plasma (0.6 ml) and red blood cell (0.6 ml) samples were immediately sent to the Center for Applied Isotope Studies at the University of Georgia (Athens, GA) for bulk carbon and nitrogen stable isotope analysis, and plasma (1 ml) was sent to Nestle Purina Analytical Labs (St. Louis, MO) for fatty acid profile testing.

### Stable Isotope Analysis

Blood samples (plasma and red blood cells) were analyzed for bulk nitrogen and carbon elemental compositions and stable isotope ratios of samples were determined using automated continuous-flow isotope ratio mass spectrometry^94^ by the Center for Applied Isotope Studies at the University of Georgia. Lipid extraction and urea removal was performed on blood samples due to the potentially conflicting role of lipids and urea in isotope analyses of elasmobranch tissues^71^. For lipid extraction, subsamples (1000 µl plasma, 500 µl red blood cells) were treated with petroleum ether (5 ml), sonicated for 15 min, and the used solution was decanted^95^. This cycle was repeated for two additional rinses until the extracted material volume remained stable. Samples were then exposed to three cycles of rinsing with ultrapure deionized water (5 ml), sonication for 15 min, and decanting for the removal of urea^88^. Sample extracts were lyophilized overnight, homogenized, weighed, and then were combusted in tin capsules using an elemental analyzer (Model NA-1500, Carlo Erba, Milan, Italy) and injected into an isotope ratio mass spectrometer (Model Delta V, Thermo Scientific, Waltham, MA). Delta (δ) values were calculated based on the following equations where Vienna Pee Dee Belemite carbonate [V-PDB] and atmospheric nitrogen [Air-N2] are standards for carbon and nitrogen, respectively:

$$\delta^{13} {\text{C}} = \left( {\frac{{\left( {\frac{{ ^{13} {\text{C}}}}{{ ^{12} {\text{C}}}}} \right){\text{ sample}}}}{{ \left( {\frac{{ ^{13} {\text{C}}}}{{ ^{12} {\text{C}}}}} \right) {\text{standard}}}} - 1} \right)$$

and

$$\delta^{15} {\text{N}} = \left( {\frac{{\left( {\frac{{ ^{15} {\text{N}}}}{{^{14} {\text{N}}}}} \right){\text{ sample}}}}{{ \left( {\frac{{ ^{15} {\text{N}}}}{{^{14} {\text{N}}}}} \right) {\text{standard}}}} - 1} \right)$$

To normalize measured isotopes to an international scale, working standards (spinach and bovine liver) were regularly analyzed across a range of C and N masses and periodically checked in-house against primary international standards (e.g., National Institute of Standards and Technology). Routine measurements were precise to within 0.15‰ for δ^13^C and δ^15^N. Data are presented as mean ± SD.

### Fatty acid analysis

Plasma samples were sent to Nestle Purina Analytical Labs (St. Louis, MO) for FA profile analysis. Briefly, lipids were extracted using the Folch method^96^, which uses a 2:1 chloroform–methanol mixture. Fatty acids were esterified with methanolic sulfuric acid, taken up in heptane, and injected on a gas chromatograph (Model 6890, Aligent Technologies, Palo Alto, CA) equipped with a non-polar Equity-1 fused silica capillary column (100 m × 0.25 mm i.d., 0.2 µm film thickness), a flame ionization detector, and a split/splitless injector. Helium was the carrier gas. The concentrations of individual FAs were calculated using an external standard method based on analysis of standards with known concentrations. The percentage for each FA was converted from the area of chromatogram peaks. Gas chromatograph results are typically subject to an error of up to ± 5% of individual component area. All FAs are expressed as percentage of total FA.

Fatty acids are denoted as X:YnZ nomenclature, where X is the number of carbon atoms, Y is the number of double bonds in the carbon chain and nZ is the position of the first double bond from the terminal methyl end of the molecule. *Cis* or *trans* configuration of the double bonds are notated with a C or T, respectively.

### Data analysis

Data were evaluated for skewness, kurtosis, and normality using Kolmogorov–Smirnov test. Data for δ^15^N and δ^13^C were not normally distributed and non-parametric Kruskal–Wallis tests were chosen for bivariate analyses along with Mann–Whitney tests to identify significantly different pair-wise comparisons. δ^15^N and δ^13^C data were power- and log-transformed, respectively, to normalize the distributions for each. Maximum likelihood linear mixed models were used to explore differences in nitrogen and carbon with sex, sampling time point, and an interaction of the two in order to determine whether sex differences varied across the sampling periods. Pairwise comparisons between the combinations of sampling time and sex were adjusted for multiple comparisons using the Šidák correction. A separate model was run for each tissue type and included a random intercept for individual animal to account for the between-subject heterogeneity. All testing was performed using Stata/SE statistical software (StataCorp, Stata Statistical Software: Release 14, College Station, TX).

Multivariate non-parametric procedures were performed to describe differences in FA profiles based on 37 FAs that were present in proportions > 0.1% of the total. FA percentages were transformed using a square root transformation to avoid over-emphasis of extreme values prior to statistical analysis. Resemblance matrices were calculated using Bray–Curtis similarity measured between samples. We measured differences in profiles using a two-factor permutational multivariate analysis of variance (PERMANOVA) with sampling time point and sex as fixed factors. Similarity percentage routines (SIMPER) were employed to identify the FAs that contributed most to dissimilarities between males and females at each sampling point. Canonical analysis of principal coordinates (CAP) was then used to further discriminate FAs between a priori groups (males and females within each sampling time point), with FAs identified by SIMPER overlaid on the CAP ordination plot. Multivariate analyses were performed using PRIMER/PERMANOVA + software (version 7, Primer-E Ltd, Aukland, New Zealand).

## Supplementary information

Supplementary Information.
