# Supplementary material for: Seasonal patterns in stable isotope and fatty acid profiles of southern stingrays (Hypanus americana) at Stingray City Sandbar, Grand Cayman
Source: Sci Rep. 2020 Nov 12;10:19753. doi: 10.1038/s41598-020-76858-w (PMC7661509; doi:10.1038/s41598-020-76858-w)
Supplement: Supplementary file 1 — Supplementary Information. [file 41598_2020_76858_MOESM1_ESM.docx]

Seasonal patterns in stable isotope and fatty acid profiles of southern stingrays (*Hypanus americana*) at Stingray City Sandbar, Grand Cayman.

**Lisa A. Hoopes^1*^, Tonya M. Clauss^2^, Nicole E. Browning^3^, Alexa J. Delaune^2^, Bradley M. Wetherbee^4,5^, Mahmood Shivji^5^, Jessica C. Harvey^6^, Guy C.M. Harvey^5^**

^1^Department of Research and Conservation and ^2^Department of Animal Health, Georgia Aquarium, Atlanta, Georgia, 30313 USA; ^3^Department of Mathematics and Science, Brenau University, Gainesville, Georgia, 30501, USA; ^4^Department of Biological Sciences, University of Rhode Island, Kingston, Rhode Island, 02881, USA; ^5^The Guy Harvey Research Institute, Halmos College of Natural Sciences and Oceanography, Nova Southeastern University, Dania Beach, Florida, USA; ^6^Guy Harvey Ocean Foundation, Grand Cayman, KY1-1005, Cayman Islands

*Corresponding author. Email: [lhoopes@georgiaaquarium.org](mailto:lhoopes@georgiaaquarium.org)

Supplemental Materials

| Table S2. Stable isotope composition of potential southern stingray prey from SE Atlantic, Gulf of Mexico, Bahamas, Cuba, and Belize based on stomach content studies. Squid values from North Atlantic and North Pacific. Data were extracted from the literature. References are 1 (Tilley et al. 2013), 2 (Abeels et al. 2012), 3 (Aguilar et al. 2008), 4 (Martinetto et al. 2006), 5 (Dittel et al. 2000), 6 (Dittel et al. 2006), 7 (Fantle et al. 1999), 8 (Winemiller et al. 2007), 9 (Peterson and Howarth 1987), 10 (Yeager and Layman 2011), 11 (Macko et al. 1984), 12 (Lerner et al. 2018), 13 (Abend and Smith 1997), 14 (Carlier et al. 2007), 15 (Chouvelon et al. 2011), 16 (Lesage et al. 2001), 17 (Carreón-Palau et al. 2013), 18 (Zhu et al. 2019). | | | | | | | | | |
| --- | --- | --- | --- | --- | --- | --- | --- | --- | --- |
|  |  | ẟ^13^C (‰) | | | ẟ^15^N (‰) | | |  |  |
| Species | Group | | Mean | SD | | Mean | SD | n | Reference |
| Lungworm, *Arenicola cristata* | Polychaete | | -8.1 | 1.1 | | 3.5 | 1.6 | 3 | 1 |
| Unidentified polychaetes | Polychaete | | -23.7 | 3.8 | | 6.7 | 1.0 | 6 | 2 |
| Unidentified polychaetes | Polychaete | | -15.8 | 1.1 | | 6.5 | 1.1 | 3 | 3 |
| Unidentified bivalves | Mollusc | | -9.7 | 1.6 | | 3.5 | 0.4 | 2 | 1 |
| Atlantic wing oyster, *Pteria colymbus* | Mollusc | | -13.6 | 0.2 | | 5.0 | 1.9 | 2 | 1 |
| Queen conch, *Aliger gigas* | Mollusc | | -12.2 | 2.2 | | 3.6 | 1.1 | 15 | 1 |
| Florida stone crab, *Menippe mercenaria* | Crustacean | | -19.5 | 1.7 | | 5.3 | 0.7 | 3 | 2 |
| Common littoral crab, *Carcinus meanus* | Crustacean | | -12.7 | 0.0 | | 8.5 | 2.2 | 4 | 4 |
| Blue crab, *Callinectes sapidus* | Crustacean | | -15.7 | 1.8 | | 9.0 | 2.0 | 22 | 4,5,6,7,8 |
| Caribbean spiny lobster, *Panulirus argus* | Crustacean | | -12.0 | 1.3 | | 5.7 | 1.2 | 43 | 1 |
| Atlantic white shrimp, *Litopenaeus setiferus* | Crustacean | | -18.9 | 2.5 | | -18.9 | 0.4 | 2 | 8,9 |
| Big claw snapping shrimp, *Alpheus heterochaelis* | Crustacean | | -22.7 | 2.4 | | 6.6 | 1.2 | 9 | 2,10 |
| Peppermint shrimp, *Lysmata wurdemanni* | Crustacean | | -20.6 |  | | 6.9 |  | 1 | 2 |
| Unidentified shrimp*, Penaeus sp.* | Crustacean | | -15.6 |  | | 12.9 |  | 1 | 11 |
| Long finned squid, *Loligo forbesii* | Squid | | -17.8 |  | | 12.5 |  | 1 | 12 |
| Longfin inshore squid, *Loligo pealei* | Squid | | -18.3 |  | | 11.9 |  | 1 | 13 |
| European squid*, Loligo vulgaris* | Squid | | -17.2 | 1.1 | | 12.2 | 3.3 | 2 | 14,15 |
| Northern shortfin squid*, Illex illecebrosus* | Squid | | -18.2 | 1.5 | | 10.5 | 3.5 | 2 | 12,16 |
| Ocean surgeon, *Acanthurus bahianus* | Teleost | | -16.1 | 0.6 | | 10.6 | 1.0 | 3 | 3 |
| Doctorfish tang, *Acanthurus chirurgus* | Teleost | | -13.7 | 1.1 | | 7.2 | 1.4 | 3 | 17,18 |
| Atlantic blue tang, *Acanthurus coeruleus* | Teleost | | -13.9 | 2.2 | | 6.2 | 1.3 | 3 | 1,18 |
| Five-band surgeonfish, *Acanthurus tractus* | Teleost | | -13.6 |  | | 5.1 |  | 1 | 18 |
| Gulf toadfish*, Opsanus beta* | Teleost | | -20.8 | 2.4 | | 7.7 | 0.6 | 8 | 2 |
| Florida blenny, *Chasmodes saburrae* | Teleost | | -24.7 | 2.3 | | 9.3 | 0.4 | 4 | 2 |
| Frillfin goby*, Bathygobius soporator* | Teleost | | -22.6 | 2.5 | | 9.7 | 1.2 | 4 | 2,10 |
| Code goby*, Gobiosoma robustum* | Teleost | | -21.6 | 3.8 | | 8.1 | 0.8 | 4 | 2 |
| Naked goby*, Gobiosoma bosc* | Teleost | | -25.1 | 0.9 | | 9.2 | 1.5 | 2 | 2 |
| Unidentified goby*, Gobiosoma sp.* | Teleost | | -24.6 |  | | 11.0 |  | 1 | 10 |
| Crested goby*, Lophogobius cyprinoides* | Teleost | | -24.4 | 1.2 | | 9.5 | 1.3 | 5 | 2,10 |
| Highfin goby, *Lupinoblennius nicholsi* | Teleost | | -23.6 | 0.3 | | 11.3 | 1.9 | 2 | 2,10 |
| Masked goby, *Coryphopterus personatus* | Teleost | | -17.1 | 0.6 | | 6.8 | 2.4 | 3 | 17,18 |
| Bridled goby, *Coryphopterus glaucofraenum* | Teleost | | -10.9 |  | | 3.9 |  | 1 | 18 |
| Cleaner goby, *Elacatinus genie* | Teleost | | -12.6 |  | | 8.9 |  | 1 | 18 |
| Bluehead wrasse, *Thalassoma bifasciatum* | Teleost | | -17.0 | 0.5 | | 7.1 | 1.3 | 4 | 3,18 |
| Slippery dick, *Halichoeres bivittatus* | Teleost | | -14.2 | 2.5 | | 9.0 | 2.1 | 4 | 3,18 |
| Yellowheaded wrasse, *Halichoeres garnoti* | Teleost | | -14.9 | 0.9 | | 9.2 | 2.0 | 4 | 3,18 |
| Mardi-gras wrasse, *Halichoeres burekae* | Teleost | | -18.0 |  | | 8.0 |  | 1 | 17 |
| Spansih hogfish, *Bodianus rufus* | Teleost | | -14.7 | 0.4 | | 9.9 | 1.2 | 2 | 17 |
| Unidentified hogfish, *Family Labridae* | Teleost | | -11.8 | 1.8 | | 7.4 | 0.7 | 7 | 1 |
| Creole wrasse, *Clepticus parrae* | Teleost | | -17.2 |  | | 5.4 |  | 1 | 18 |
| Clown wrasse, *Halichoeres maculipinna* | Teleost | | -13.7 |  | | 6.7 |  | 1 | 18 |
| Rainbow wrasse, *Halichoeres pictus* | Teleost | | -17.4 |  | | 4.5 |  | 1 | 18 |
| Lionfish, Ppterois volitans | Teleost | | -11.4 | 0.3 | | 8.5 | 0.1 | 4 | 1 |

Figure S1. Map of Grand Cayman Island highlighting the Stingray City Sandbar site. This map was created with ArcGIS Enterprise 10.7.1 (https://enterprise.arcgis.com).


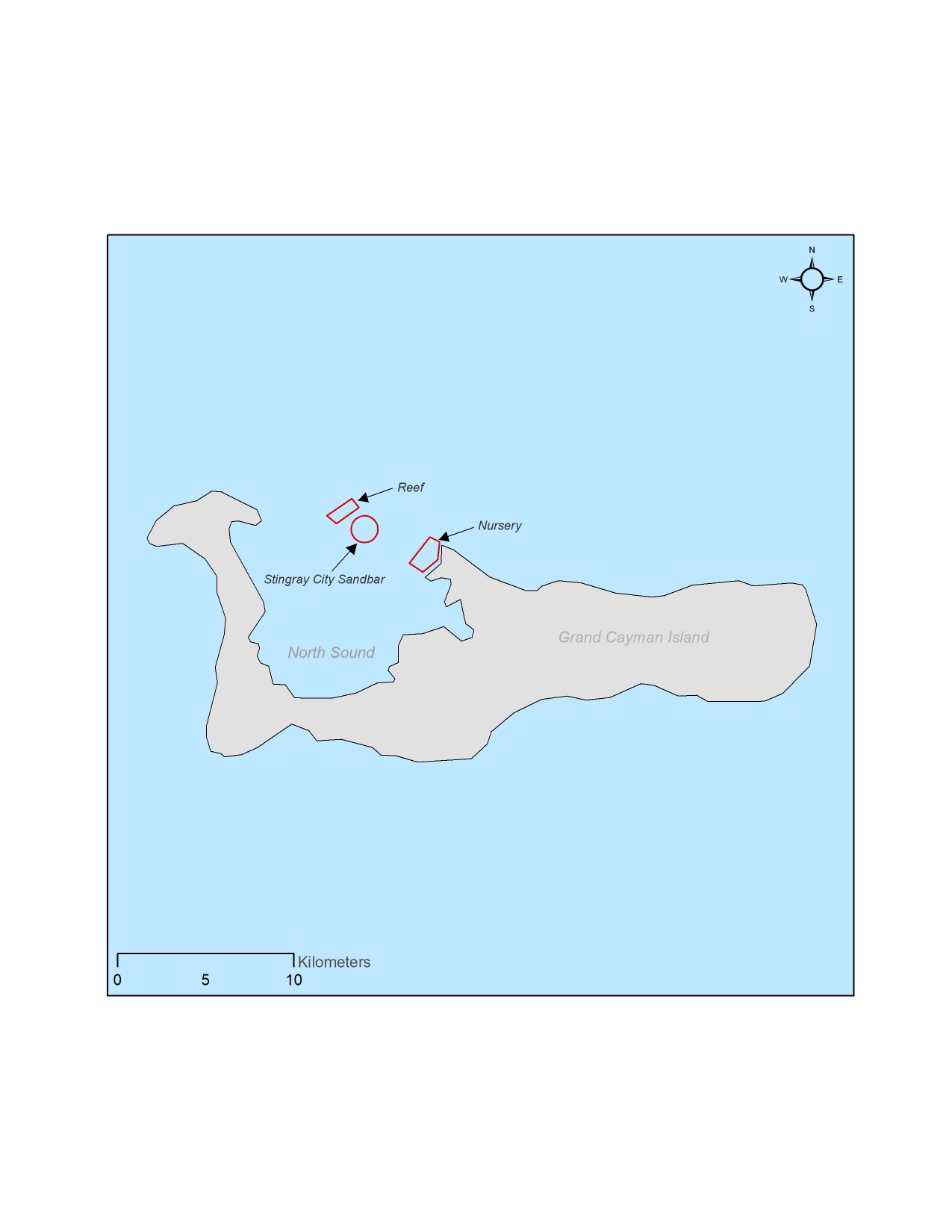


References

1. Tilley, A., López-Angarita, J. & Turner, J.R. Diet reconstruction and resource partitioning of a Caribbean marine mesopredator using stable isotope Bayesian modeling. *PLoS One* **8(11)**, e79560 [doi.org/10.1371/journal.pone.0079560](https://doi.org/10.1371/journal.pone.0079560) (2013).

2. Abeels, H.A., Loh, A.N. & Volety, A.K. Trophic transfer and habitat use in oyster *Crassostrea virginica* reefs in southwest Florida identified by stable isotope analysis. *Mar. Ecol. Prog. Ser.* **462**, 125-142 (2012).

3. Aguilar, C., González-Sansón, G., Faloh, I. & Curry, R.A. Spatial variation is table isotopes (δ^13^C and δ^15^N) in marine fish along the coast of Havana City: evidence of human impacts from harbor and river waters. *J. Coast. Res.* **24**, 1281-1288 (2008).

4. Martinetto, P., Teichberg, M. & Valiela, I. Coupling of estuarine and benthic food webs to land-derived nitrogen sources in Waquoit Bay, Massachusetts, USA. *Mar. Ecol. Prog. Ser.* **307**, 37-48 (2006).

5. Dittel, A.I., Epifanio, C.E., Schwalm, S.M., Fantle, M.S. & Fogel, M.L. Carbon and nitrogen sources for juvenile blue crabs *Callinectes sapidus* in coastal wetlands. *Mar. Ecol. Prog. Ser.* **194**, 103-112 (2000).

6. Dittel, A.I., Epifanio, C.E. & Fogel, M.L. Trophic relationships of juvenile blue crabs in estuarine habitats. *Hydrobiologica* **568**, 379-390 (2006).

7. Fantle, M.S., Dittel, A.I., Schwalm, S.M., Epifanio, C.E. & Fogel, M.L. A food web analysis of the juvenile blue crab, *Callinectes sapidus*, using stable isotopes in whole animals and individual amino acids. *Oecologica* **120**, 416-426 (1999).

8. Winemiller, K.O., Akin, S. & Zeug, S.C. Production sources and food web structure of a temperate tidal estuary: integration of dietary and stable isotope data. *Mar. Ecol. Prog. Ser*. **343**, 63-76 (2007).

9. Peterson, B.J. & Howarth, R.W. Sulfur, carbon, and nitrogen isotopes used to trace organic matter flow in the salt-marsh estuaries of Sapelo Island, Georgia. *Limnol. Oceanogr.* **32**, 1195-1213 (1987).

10. Yeager, L.A. & Layman, C.A. Energy flow to two abundant consumers in a subtropical oyster reef food web. *Aquat. Ecol*. **45**, 267-277 [doi.org/10.1007/s10452-011-9352-1](http://dx.doi.org/10.1007/s10452-011-9352-1)

(2011).

11. Macko, S., Entzeroth, L. & Parker, P.L. Regional differences in nitrogen and carbon isotopes on the Continental Shelf of the Gulf of Mexico. *Naturwissenschaften*, **71**, 374-375 (1984).

12. Lerner, J.E. *et al.* Evaluating the use of stable isotope analysis to infer the feeding ecology of a growing US gray seal (*Halichoerus grypus*) population. *PLoS One* **13(2)**, e0192241 [doi.org/10.1371/journal.pone.0192241](https://doi.org/10.1371/journal.pone.0192241) (2018).

13. Abend, A.G. & Smith, T.D. Differences in stable isotope ratios of carbon and nitrogen between long-finned pilot whales (*Globicephala melas*) and their primary prey in the western north Atlantic. *ICES J. Mar. Sci.* **54**, 500–503 (1997).

14. Carlier, A., Riera, P., Amouroux, J.-M., Bodiou, J.-Y. & Grémare, A. Benthic trophic network in the Bay of Banyuls-sur-Mer (northwest Mediterranean, France): an assessment based on stable carbon and nitrogen isotope analysis. *Estuar. Coast. Shelf S.* **72**, 1-15 (2007).

15. Chouvelon, T. *et al.* Inter-specific and ontogenetic differences in δ^13^C and δ^15^N values and Hg and Cd concentrations in cephalopods. *Mar. Ecol. Prog. Ser.* **433**, 107-120 (2011).

16. Lesage, V., Hammill, M.O. & Kovacs, K.M. Marine mammals and the community structure of the Estuary and Gulf of St Lawrence, Canada: evidence from stable isotope analysis. *Mar. Ecol. Prog. Ser.* **210**, 203-221 (2001).

17. Carreón-Palau, L., Parrish, C.C., del Angel-Rodríguez, J.A., Pérez-España, H. & Aguiñiga-Garcia, S. Revealing organic carbon sources fueling a coral reef food web in the Gulf of Mexico using stable isotopes and fatty acids. *Limnol. Oceanogr*. **58**, 593-612 (2013).

18. Zhu, Y., Newman, S.P., Reid, W.D.K & Polunin, N.V.C. Fish stable isotope community structure of a Bahamian coral reef. *Mar. Biol.* **166**, [doi.org/10.1007/s00227-019-3599-9](https://doi.org/10.1007/s00227-019-3599-9) (2019).
